# Liver Cancer Detection by a Simple, Inexpensive and Effective Immunosensor with Zinc Oxide Nanoparticles

**DOI:** 10.3390/s151129408

**Published:** 2015-11-20

**Authors:** Congo Tak-Shing Ching, Nguyen van Hieu, Teng-Yun Cheng, Lin-Shien Fu, Tai-Ping Sun, Ming-Yen Liu, Su-Hua Huang, Yan-Dong Yao

**Affiliations:** 1 Department of Electrical Engineering, National Chi Nan University, Nantou 54561, Taiwan; E-Mails: s103323905@mail1.ncnu.edu.tw (T.-Y.C.); president.sun@nkut.edu.tw (T.-P.S.); s102323520@mail1.ncnu.edu.tw (M.-Y.L.); 2 Department of Photonics and Communication Engineering, Asia University, Taichung 41354, Taiwan; 3 Department of Physics and Electronic Engineering, University of Science (Vietnam National University of Hochiminh City), District 5, Hochiminh City 999100, Vietnam; E-Mail: nvhieu@hcmus.edu.vn; 4 Department of Pediatrics, Taichung Veterans General Hospital, Taichung 40705, Taiwan; E-Mail: lsfu@vghtc.gov.tw; 5 Department of Electronics Engineering, Nan Kai University of Technology, Nantou 54243, Taiwan; 6 Department of Biotechnology, Asia University, Taichung 41354, Taiwan; 7 Division of Science & Technology, Hong Kong Community College, Hong Kong, China; E-Mail: ydyao@hkcc-polyu.edu.hk

**Keywords:** liver cancer, DCP, immunosensor, screening, nanoparticle

## Abstract

Regular monitoring of blood α-fetoprotein (AFP) and/or carcino-embryonic antigen (CEA) levels is important for the routine screening of liver cancer. However, AFP and CEA have a much lower specificity than des-γ-carboxyprothrombin (DCP) to detect liver cancer. Therefore, the study reported here was designed, to develop a screen-printed DCP immunosensor incorporating zinc oxide nanoparticles, for accurate determination of DCP. The designed immunosensor shows low detection limits for the detection of DCP: 0.440 ng/mL (based on impedance measurement), 0.081 ng/mL (based on real part of impedance measurement) and 0.078 ng/mL (based on imaginary part of impedance measurement), within the range of 3.125 ng/mL to 2000 ng/mL. In addition, there was little interference to DCP determination by molecules such as Na^+^, K^+^, Ca^2+^, Cl^−^, glucose, urea, and uric acid. It is therefore concluded that the DCP immunosensor developed and reported here is simple, inexpensive and effective, and shows promise in the rapid screening of early-stage liver cancer at home with a point-of-care approach.

## 1. Introduction

Cancers was top of the leading causes of death (28.4%) in Taiwan in 2012, as reported by the Ministry of Health and Welfare (Taiwan) [1,2]. Within these cancer cases, liver cancer mortality was the second leading cause of death (18.6%) [1,2], and the prevalence of liver cancer has risen from 15.3% in 1981 to 34.9% in 2012 [1,2]. It was estimated that 34.9 out of 100,000 people died because of liver cancer [1,2]. Moreover, early-stage liver cancer cases are often undetected, resulting in late diagnosis, treatment and management, exerting a heavy burden on the national health insurance system.

Clinically, liver cancer can be screened for by using the α-fetoprotein (AFP) test and carcino-embryonic antigen (CEA) test [3,4,5,6]. However, benign liver diseases can also cause an increase in blood AFP levels, and CEA is commonly used for screening several forms of cancer, not specifically liver cancer [7,8]. Therefore, both the use of AFP and CEA could potentially lead to misdiagnosis. Moreover, AFP and CEA screenings are restricted to the hospital or clinic, not residential settings. It is thus possible that people at higher risk of liver cancer may be deterred from taking regular AFP and CEA tests on account of travelling, missing the window of early detection and treatment.

On the ground of the previously mentioned issues, the study reported here aims to develop a simple screen-printed des-γ-carboxyprothrombin (DCP) immunosensor incorporating zinc oxide nanoparticles (ZnO-NPs), for accurate determination of DCP. This is further supported by the recent publication of DCP being a new biomarker, possessing approximately 70% sensitivity and close to 100% specificity for liver cancer detection [9]. Basing on the ultra-high specificity of DCP for liver cancer detection, successful development of a simple and highly-sensitive DCP immunosensor, would provide a new tool for early detection of liver cancer at home, using a point-of-care approach. With this new sensor, the present requirement of sophisticated instruments needed to detect very low levels of DCP, would be bypassed. To the best of the authors’ knowledge, there is no reported research on a DCP immunosensor at present, and the nanomaterials deployed in this study can enhance sensitivity of biosensors [10,11,12,13]. ZnO-NP is good source for immobilization of proteins due to its strong adsorption ability (high isoelectric point ~9.5), good biocompatibility, and high electron communication features [14,15,16,17,18,19,20,21,22]. Many studies have been reported the use of ZnO-NP to fabricate biosensors. For example, Ren *et al.* [23] reported that ZnO-NP can enhance (25-fold) the current response of a glucose biosensor because of the large surface area of ZnO-NP and the surface of ZnO-NP can facilitate the enzyme immobilization [24]. Hence, the study reported here aims to develop a DCP immunosensor incorporating ZnO-NP, to improve the sensitivity for detection of low-level of DCP.

## 2. Materials and Methods

### 2.1. Chemicals and Reagents

Commercial chemicals and reagents without further purification were used in this study. Phosphate-buffered saline (PBS), bovine serum albumin (BSA), glutaraldehyde and 20 nm zinc oxide nanoparticle were from Sigma Chemical (St Louis, MO, USA); DCP antigen and antibody were from AllBio Science Inc. (Taiching, Taiwan); Graphite and silver pastes were purchased from Advanced Conductive Materials (Atascadero, CA, USA); Epoxy (EPO-TEK^®^ 509FM-1) from Epoxy Technology (Billerica, MA, USA), and polyethylene terephthalate (PET) sheet from 3M (Taipei, Taiwan). Deionized water (resistivity ≥ 18 MΩ·cm) used for all preparations, was purified by a Milli-Q UFplus System (Millipore, Bedford, MA, USA).

### 2.2. Equipment

An impedance analyzer (Precision Impedance Analyzer WK6420C, Wayne Kerr Electronics Ltd., London, UK), was used for measurements of impedance (Z) spectrum, real part of impedance (Z') spectrum and imaginary part of impedance (Z") spectrum.

### 2.3. Fabrication of DCP Immunosensor

**Figure 1:**
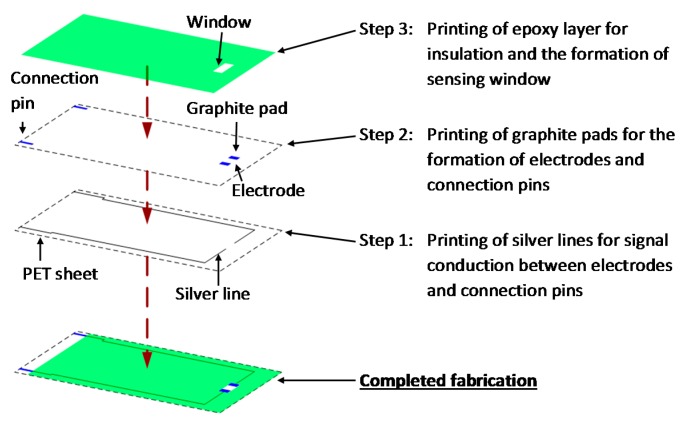
Fabrication procedure of the designed DCP immunosensor, using polyethylene terephthalate (PET) as substrate. Each layer of printing was allowed to dry at 100 °C for 30 min.

A screen printing technique (screen mesh size = 390 counts per inch; screen emulsion thickness = 25 µm), was employed to construct the sensor used in this study. The fabrication procedure according to a published procedure [25] is schematically shown in Figure 1. In brief, each sensor has three different screen printing layers, each formed in succession on a clear PET sheet. Each layer was allowed to dry at 100 °C for 30 min. The first printing layer consists of silver lines for signal conduction. The second layer of graphite pads provided a base for antibody immobilization and the formation of connection pins; while the third layer being the insulating shroud of epoxy used for insulation and formation of a testing well. 

DCP antibody immobilization was achieved by pipetting a mixture (2 µL) of glutaraldehyde (2.5%) and ZnO-NP (0.2 mg/100 mL) into the window, formed by epoxy insulating shroud on the sensor. After 1 min, a mixture (4 µL) of DCP antibody (2 µg/mL, 2 µL) and BSA (0.1 M, 2 µL) was subsequently pipetted into the window on the sensor, and allowed to cross-link, before cooling the sensor overnight at 4 °C.

### 2.4. Measurements of the Immunosensor Response to DCP

All Z, Z' and Z" spectrum measurements were carried out at room temperature (~25 °C), with a measurement frequency ranging from 300 Hz to 5 MHz, with 100 frequency points per logarithmic decade within this frequency range. Amplitude of the perturbing wave was set to 100 mV. 

Measurements were obtained by connecting the DCP immunosensor to the impedance analyzer. One minute after pipetting PBS (10 µL, 25 mM, pH 7.0) onto the immunosensor, Z, Z' and Z" spectra of the PBS (Z_PBS_, Z'_PBS_ and Z"_PBS_, respectively) were then captured. Then with the PBS removal, 10 µL DCP (3.125, 6.25, 125 and 2000 ng/mL) was pipetted onto the immunosensor. After 30 min, the DCP was removed and the immunosensor immersed in and gently washed with fresh PBS (25 mM, pH 7.0). A fresh PBS (10 µL, 25 mM, pH 7.0) was consequently pipetted onto the immunosensor, the Z, Z' and Z" spectra were captured after 1 minute. These measurements were denoted as the Z, Z’ and Z" spectra of the DCP (Z_DCP_, Z'_DCP_ and Z"_DCP_, respectively).

The Z responses of the immunosensor to DCP were calculated, by subtracting Z_DCP_ from Z_PBS_ (*i.e*., Z_DCP_ − Z_PBS_) for DCP of various concentrations. The Z' responses of the immunosensor to DCP were calculated, by subtracting Z'_DCP_ from Z'_PBS_ (*i.e*., Z'_DCP_ − Z'_PBS_) for DCP of various concentrations. The Z" responses of the immunosensor to DCP were calculated, by subtracting Z"_DCP_ from Z"_PBS_ (*i.e*., Z"_DCP_ − Z"_PBS_) for DCP of various concentrations.

## 3. Results and Discussion

**Figure 2:**
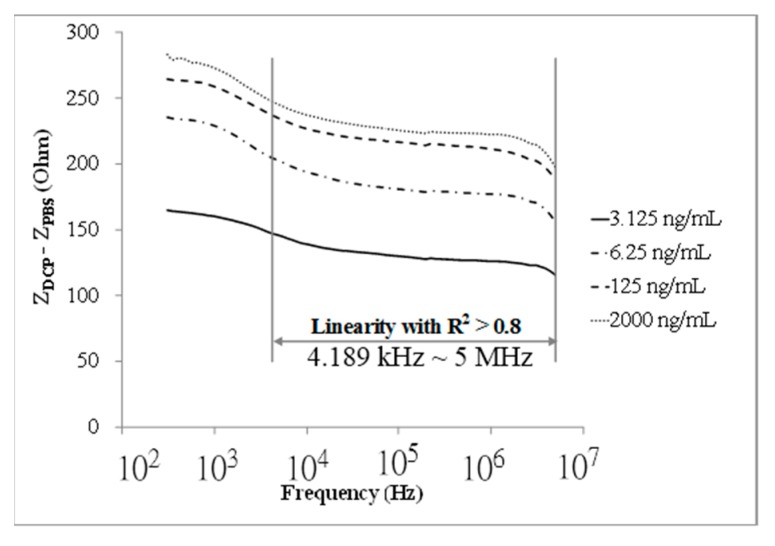
The impedance (Z) response of the immunosensor to DCP at various concentrations (3.125, 6.25, 125, 2000 ng/mL) within a frequency range of 300–5 MHz.

The Z responses (*i.e.*, Z_DCP_ − Z_PBS_) of the designed immunosensor to DCP at various concentrations (3.125–2000 ng/mL) within the frequency range of 300 Hz–5 MHz, are shown in Figure 2. A specific frequency range (4.189 kHz–5 MHz) was found, in which a good linear (correlation coefficient, R^2^ > 0.8) response range can be obtained. 

**Figure 3:**
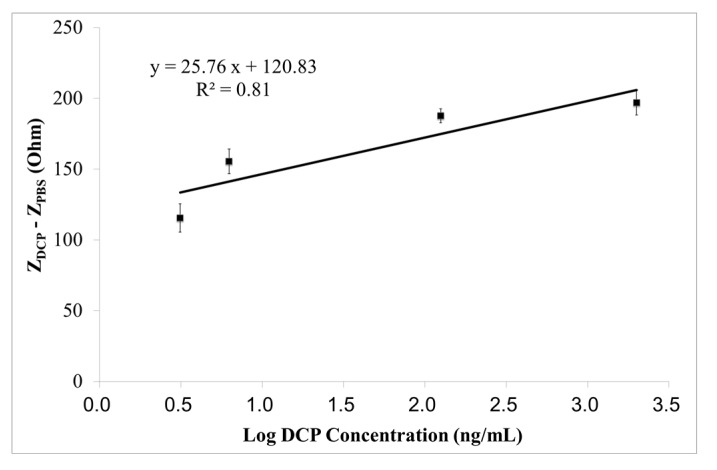
The linear calibration curve (Z_DCP_ − Z_PBS_ *vs.* Log DCP concentration) of the immunosensor on measuring DCP (3.125–2000 ng/mL) at the optimum measuring frequency of 5 MHz, within the specific frequency range (4.189–5 MHz). Results are expressed in mean ± SD (n = 5).

The linear calibration curve of the designed immunosensor for measuring DCP at an optimum frequency of 5 MHz, is shown in Figure 3. A good linear (R^2^ = 0.81) response was found, with sensitivity of 25.76 Ω/Log (ng/mL) and limit of detection (LOD) of 0.440 ng/mL, at a signal-to-noise ratio (S/N ratio) of 3.

**Figure 4:**
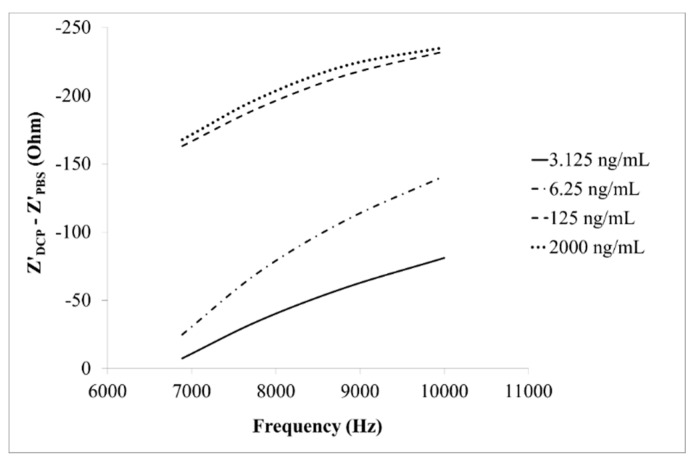
The real part of impedance (Z') response of the immunosensor to DCP of various concentrations (3.125, 6.25, 125, 2000 ng/mL) within a specific frequency range (6.887–10 kHz).

The real part of Z' response (*i.e*., Z'_DCP_ − Z'_PBS_) of the designed immunosensor to DCP of various concentrations (3.125–2000 ng/mL) within a specific frequency range (6.887–10 kHz), is shown in Figure 4. It was within this range of frequency, a good linear (R^2^ > 0.8) response range was observed. 

**Figure 5:**
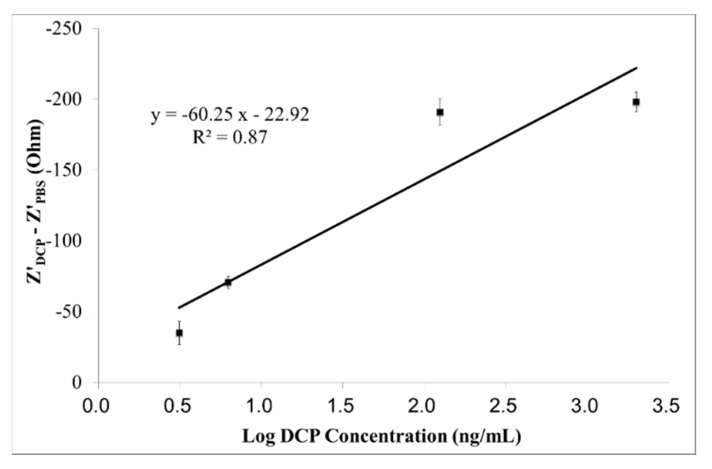
The calibration curve (Z'_DCP_ − Z'_PBS_ *vs.* Log DCP concentration) of the immunosensor on measuring DCP (3.125–2000 ng/mL) at the optimum measuring frequency of 7.799 kHz, within the specific frequency range (6.887–10 kHz). Results are expressed in mean ± SD (n = 5).

The linear calibration curve of the immunosensor, for measuring DCP at the optimum measuring frequency of 7.799 kHz, is shown in Figure 5. The designed immunosensor shows a good linear (R^2^ = 0.87) response with the sensitivity of −60.25 Ω/Log (ng/mL) and LOD of 0.081 ng/mL (S/N ratio = 3).

**Figure 6:**
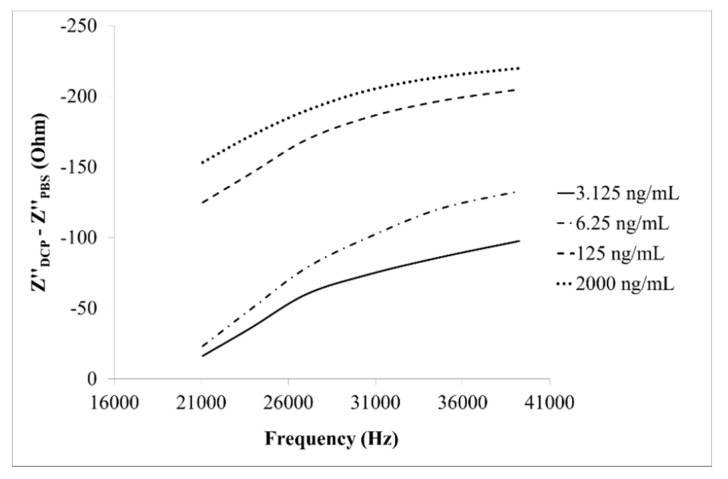
The imaginary part of impedance (Z") response of the immunosensor to DCP of various concentrations (3.125, 6.25, 125, 2000 ng/mL) within a specific frequency range (21.080–39.244 kHz).

The imaginary part of Z" response (*i.e*., Z"_DCP_ − Z"_PBS_) of the designed immunosensor to DCP of various concentrations (3.125–2000 ng/mL) within a specific frequency range (21.080–39.244 kHz), is displayed in Figure 6. An excellent linear (R^2^ > 0.9) response range was observable. 

**Figure 7:**
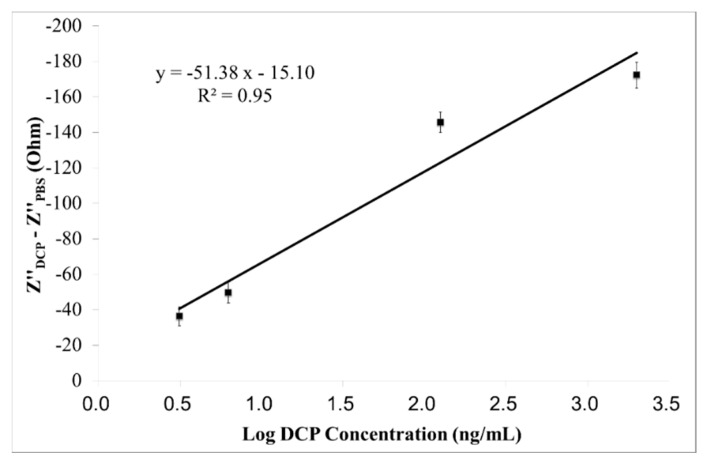
Linear calibration curve (Z"_DCP_ − Z"_PBS_ *vs.* Log DCP concentration) of the immunosensor on measuring DCP (3.125–2000 ng/mL) at the optimum measuring frequency of 23.870 kHz, within the specific frequency range (21.080–39.244 kHz). Results are expressed in mean ± SD (n = 5).

The linear calibration curve of the immunosensor on measuring DCP at the optimum measuring frequency of 23.870 kHz, is displayed in Figure 7. The designed immunosensor shows an excellent linear (R^2^ = 0.95) response, with sensitivity of −51.38 Ω/Log (ng/mL) and LOD of 0.078 ng/mL (S/N ratio = 3).

Repeatability and reliability tests have been conducted (Table 1), to evaluate the reliability of the immunosensor on DCP determination. The immunosensor was evaluated by measuring Z" response of the immunosensor to DCP (3.125 ng/mL), at an optimum measuring frequency of 23.870 kHz. 

**Table 1:** The performance of the DCP immunosensor.

Performance of the DCP Immunosensor		Actual
Linear Range		3.125–2000 ng/mL
Sensitivity	Based on Z measurement	25.76 Ω/Log (ng/mL)
	Based on Z' measurement	−60.25 Ω/Log (ng/mL)
	Based on Z" measurement	−51.38 Ω/Log (ng/mL)
Linearity	Based on Z measurement	R^2^ = 0.81
	Based on Z' measurement	R^2^ = 0.87
	Based on Z" measurement	R^2^ = 0.95
Limit of Detection (S/N ratio = 3)	Based on Z measurement	0.440 ng/mL
	Based on Z' measurement	0.081 ng/mL
	Based on Z" measurement	0.078 ng/mL
SFR	Based on Z measurement	4.189 kHz–5 MHz
	Based on Z' measurement	6.887–10 kHz
	Based on Z" measurement	21.080–39.244 kHz
OMF	Based on Z measurement	5.00 MHz
	Based on Z' measurement	7.799 kHz
	Based on Z" measurement	23.870 kHz
Repeatability	Coefficients of variations	3.69%
Reliability	Intra-rater reliability (ICC 3,k)	0.97
	Inter-rater reliability (ICC 2,k)	0.95
Stability (4 °C storage in a dry & dark condition for 20 days)		Retaining 89% of its initial value

Where SFR, OMF and ICC are the specific frequency range, optimum measuring frequency and intraclass correlation coefficients, respectively.

Repeatability tests showed that the immunosensor is reliable enough to determine the concentrations of DCP, with a coefficient of variation of 3.69%. The intra-class correlation coefficient (ICC) is a measure, used to quantify the reproducibility of a variable, as well as a measure of the homogeneity within groups of replicate measurements, relative to the total variation between groups. It has been suggested that ICC values should be above 0.75 for good reliability in general [26]. Moreover, ICC values should exceed 0.90 to ensure reasonable validity for many clinical measurements’ reliability [26]. Outcome of reliability test confirms the excellent reliability and validity of the immunosensor, with the ICC(3,k) and ICC(2,k) values being 0.97 and 0.95, respectively (Table 1).

Based on the findings shown in Figure 3, Figure 5 and Figure 7 and Table 1, the Z" response of the immunosensor to DCP is the best approach among the other two (*i.e*., Z and Z' responses), for the screening of early-stages liver cancer, as it provides the lowest LOD (0.078 ng/mL) and the highest linearity (R^2^ = 0.95). 

Stability of the immunosensor under dry and dark conditions at 4 °C, was evaluated over a 20 day period. The Z" response of the immunosensor to DCP (6.25 ng/mL) at the optimum measuring frequency of 23.870 kHz, was about 89% of its initial value. This finding suggests a good stability of the immunosensor, possibly due to the use of glutaraldehyde and BSA to crosslink the antibody onto the sensor. This crosslinking structure possibly provides a good micro-environment for the maintenance of the functional activity of antibodies. 

An interference test has been conducted, to evaluate the selectivity of the immunosensor on DCP determination (Table 2). The immunosensor was evaluated by measuring the Z" response of the immunosensor to DCP (4–2000 ng/mL), in the presence of high levels of Na^+^, K^+^, Ca^2+^, Cl^−^, glucose, urea, and uric acid (1 μM for all interferences, and 5 mM for glucose) in BSA. All measurements were taken at the optimum measuring frequency of 23.870 kHz. As shown in Table 2, the recovery of the immunosensor on DCP (4–2000 ng/mL) determination is greater than 95%. Therefore, the selectivity of the immunosensor is acceptable. In addition, this finding suggests that the immunosensor is effective for the quantitative determination of DCP in most real samples (*i.e*., BSA with interferences).

**Table 2:** Measurements of DCP with interferences of Na^+^, K^+^, Ca^2+^, Cl^−^, glucose, urea, and uric acid at high concentrations (1 μM for all interferences, and 5 mM for glucose) in BSA. Linear calibration curve (Z"_DCP_ − Z"_PBS_ *vs.* Log DCP_concentration_) in Figure 7 was used, with the linear regression equation of ∆Z = −51.38 × Log DCP_concentration_ − 15.10. All measurements were conducted at the optimum measuring frequency of 23.870 kHz.

Standard DCP (ng/mL)	Actual (ng/mL)	Recovery (%)	Relative Error (%)
4	4.15	96.2	3.8
20	20.82	95.9	4.1
200	206.41	96.8	3.2
2000	2094.13	95.3	4.7

Where Na^+^, K^+^, Ca^2+^, Cl^−^, ∆Z, and DCP_concentration_ are the sodium ion, potassium ion, calcium ion, chloride ion, change of imaginary part of impedance (*i.e*., Z"_DCP_ − Z"_PBS_) and DCP concentration, respectively.

## 4. Conclusions

A simple, inexpensive and effective DCP immunosensor incorporating zinc oxide nanoparticles, was successfully designed and developed. The Z" response of the immunosensor to DCP is an effective tool for early liver cancer detection, as it provides the lowest LOD (0.078 ng/mL), the highest linearity (R^2^ = 0.95) and good sensitivity (−51.38 Ω/Log(ng/mL)). The DCP immunosensor has a linear working range of 3.125–2000 ng/mL. Therefore, a new screening tool is proposed and tested in this study, possessing the benefits of ease of use at home, short testing time, affordability and being a good tool for a possible point-of-care approach.
